# Psychosocial interventions for patients with advanced cancer – a systematic review of the literature

**DOI:** 10.1038/sj.bjc.6602103

**Published:** 2004-08-17

**Authors:** R J Uitterhoeve, M Vernooy, M Litjens, K Potting, J Bensing, P De Mulder, T van Achterberg

**Affiliations:** 1 University Medical Centre Nijmegen, Division of Internal Diseases 166, PO Box 9101, 6500 HB Nijmegen, The Netherlands; 2 Nijmegen University, The Centre for Quality of Care Research, Nijmegen, The Netherlands; 3 Netherlands Institute for Health Services Research, Research Institute for Psychology and Health, Utrecht, The Netherlands; 4 University Medical Centre Nijmegen, Department of Medical Oncology, Nijmegen, The Netherlands; 5 University Medical Centre Nijmegen, Department of Nursing Science, Nijmegen, The Netherlands

**Keywords:** advanced cancer, palliative care, systematic review, psychosocial interventions, behaviour therapy, quality of life

## Abstract

Advanced cancer is associated with emotional distress, especially depression and feelings of sadness. To date, it is unclear which is the most effective way to address these problems. This review focuses on the effects of psychosocial interventions on the quality of life (QoL) of patients with advanced cancer. It was hypothesised that patients will benefit from psychosocial interventions by improving QoL, especially in the domain of emotional functioning. The review was conducted using systematic review methodology involving a systematic search of the literature published between 1990 and 2002, quality assessment of included studies, systematic data extraction and narrative data synthesis. In all, 10 randomised controlled studies involving 13 trials were included. Overall interventions and outcome measures across studies were heterogeneous. Outcome measures, pertaining to the QoL dimension of emotional functioning, were most frequently measured. A total of 12 trials evaluating behaviour therapy found positive effects on one or more indicators of QoL, for example, depression. The results of the review support recommendation of behaviour therapy in the care of patients with advanced cancer.

In 1998, approximately 60 000 new cancer patients were diagnosed in the Netherlands (Visser *et al*, 2002). In that same year, 37 000 patients died of this disease (Visser *et al*, 2002). About half of all patients cannot be cured and receive treatment with a palliative intent.

Clearly, the emotional impact of a cancer diagnosis is devastating and characterised by shock, disbelief, anger, anxiety, depression and difficulty in performing activities of daily living. A similar response occurs at each transitional point of the disease, that is, beginning treatment, recurrence, treatment failure and disease progression (Pasacreta and Pickett, 1998). Although it is obvious that many patients with cancer experience emotional distress, van't Spijker *et al* (1997) found that percentages for depression varied from 0 to 46%, for anxiety from 0.9 to 49% and for general psychological distress from 5 to 50%. These data do not refer to patients in a specific stage of cancer, which may account for the wide variation in prevalence rates. Less variation in prevalence rates of emotional distress is found in patients with advanced disease. In this population, emotional distress and depression, in particular, appear to be a common problem (Zabora *et al*, 1997; Massie and Popkin, 1998). Hotopf *et al* (2002) estimated that the prevalence of depression ranged from 15% for major depression to at least 30% for all depressive disorders (including minor depression).

Moreover, several studies (Slevin *et al*, 1996; Sanson-Fisher *et al*, 2000; Soothill *et al*, 2001) report that patients in an advanced stage of the disease have high levels of psychosocial needs that are not properly met. Professional caregivers appear to be selective in their receptiveness of patients' needs, focus on physical problems and to a much lesser extent on emotional problems and psychosocial needs. This implies that psychological problems and emotional needs are not adequately assessed (Heaven and Maguire, 1997; Sanson-Fisher *et al*, 2000; Fallowfield *et al*, 2001) and consequently addressed (Wilkinson, 1991; Dennison, 1995; Ford *et al*, 1996; Maguire *et al*, 1996; Heaven and Maguire, 1997; Suchman *et al*, 1997; Maguire, 1999; Osse *et al*, 2000; Andersen and Adamsen, 2001; Uitterhoeve *et al*, 2003).

In the last decade, several systematic reviews (Trijsburg *et al*, 1992; Devine and Westlake, 1995; Meyer and Mark, 1995; Sheard and Maguire, 1999; Barsevick *et al*, 2002; Newell *et al*, 2002) were published about the effectiveness of psychosocial interventions for a general population of patients with cancer. Each review had somewhat different objectives, for example, outcomes of interest between reviews ranged from all possible psychosocial outcomes to survival and immune outcomes. Similarly, each review employed different inclusion criteria and controlled for study quality in different ways. Despite these differences, overall it appears that psychosocial interventions to some extent may help patients with cancer. Especially, patients identified as either suffering from or being at high risk for psychological distress seem to benefit (Sheard and Maguire, 1999). None of the mentioned reviews (Trijsburg *et al*, 1992; Devine and Westlake, 1995; Meyer and Mark, 1995; Sheard and Maguire, 1999; Newell *et al*, 2002; Barsevick *et al*, 2002), however, explicitly focused on the effects of psychosocial interventions in patients with cancer in an advanced stage of the disease.

Hence, a systematic review of the literature on the effectiveness of psychosocial interventions in patients with advanced cancer is conducted. It is hypothesised that patients with advanced cancer will benefit from psychosocial interventions by improving quality of life (QoL), especially in the domain of emotional functioning. The aim of this systematic review was to identify and examine all known controlled studies published between 1990 and 2002 pertaining to the efficacy of psychosocial interventions on the QoL of adult cancer patients in an advanced stage of the disease.

## MATERIALS AND METHODS

### Search strategy

First, computerised databases of Medline (1989–2002), PsycInfo (1988–2002) and Cinahl (1982–2002) were searched using the following procedure. Subject-specific keywords used to describe patients and interventions relevant to this review were selected by using the thesaurus function of the databases. The selected subject-specific keywords for patients and psychosocial interventions were separately combined (using Boolean operator ‘OR’) with relevant free text words. The two searches were then combined (using Boolean operator ‘AND’) to limit the search to studies with cancer patients in an advanced stage of the disease, which mention psychosocial or any of the approximate synonyms for psychosocial interventions. Next, the above combined search was then, respectively, combined (using Boolean operator ‘AND’) with a database specific methodological filter adapted from Robinson and Dickersin (2002) limiting the search to controlled studies. The search was then limited to papers published between 1990 and 2002 (Table 1

**Table 1:** Search strategy

**Medline (1990–2002)**	**Cinahl (1990–2002)**	**PsycInfo (1990–2002)**
((((‘Neoplasms-’/all subheadings in MIME,MJME) or (cancer^*^ in ab) or (tumor^*^ in ab) or (tumour^*^ in ab) or (malign^*^ in ab) or (oncolog^*^ in ab)) and ((‘Palliative-Care’/all subheadings in MIME,MJME) or (‘Terminal-Care’/all subheadings in MIME,MJME) or (‘Hospice-Care’/all subheadings in MIME,MJME) or (‘Terminally-Ill’/all subheadings in MIME,MJME) or (incurable in ab) or (incurable in ti) or (advanced in ab) or (advanced in ti) or (palliat^*^ in ab) or (palliat^*^ in ti) or (terminal^*^ in ab) or (terminal^*^ in ti))) and ((explode ‘Psychotherapy-’/all subheadings in MIME,MJME) or (‘Patient-Education’/all subheadings in MIME,MJME) or (‘Cognitive-Therapy’/all subheadings in MIME,MJME) or (explode ‘Behavior-Therapy’/all subheadings in MIME,MJME) or (explode ‘Adaptation-Psychological’/all subheadings in MIME,MJME) or (‘Counseling-’/all subheadings in MIME,MJME) or (‘Social-Support’/all subheadings in MIME,MJME) or (psychosocial in ab) or (psychosocial in ti))) and (((Randomized-Controlled-Trial in pt) or (Controlled-Clinical-Trial in pt) or (randomized controlled trials in MIME,MJME) or (random allocation in MIME,MJME) or (double-blind method in MIME,MJME) or (single-blind method in MIME,MJME) or (Clinical-Trial in pt) or (clinical trials in MIME,MJME) or (‘clinical trial’) or ((singl^*^ or doubl^*^ or trebl^*^ or tripl^*^) and (mask^*^ or blind^*^)) or (‘latin square’) or (placebos in MIME,MJME) or placebo^*^ or random^*^ or (research design in MIME,MJME) or (comparative study in MIME,MJME) or (evaluation studies in MIME,MJME) or (follow-up studies in MIME,MJME) or (prospective studies in MIME,MJME) or (cross-over studies in MIME,MJME) or control^*^ or prospective^*^ or volunteer^*^) not ((animal in MIME,MJME) not (human in MIME,MJME)))	(((‘Neoplasms-’/all topical subheadings/all age subheadings in DE) or (cancer in ab) or (tumor^*^ in ab) or (tumour^*^ in ab) or (malign^*^ in ab) or (oncolog^*^ in ab)) and ((explode ‘Terminal-Care’/all topical subheadings/all age subheadings in DE) or (‘Terminally-Ill-Patients’/all topical subheadings/all age subheadings in DE) or (incurable in ab) or (incurable in ti) or (advanced in ab) or (advanced in ti) or (palliat^*^ in ab) or (palliat^*^ in ti) or (terminal^*^ in ab) or (terminal^*^ in ti)))) and (((explode ‘Psychotherapy-’/all topical subheadings/all age subheadings in DE) or (explode ‘Behavior-Therapy’/all topical subheadings/all age subheadings in DE) or (‘Coping-’/all topical subheadings/all age subheadings in DE) or ((‘Caregiver-Support’/all topical subheadings/all age subheadings in DE) or (‘Support-Groups’/all topical subheadings/all age subheadings in DE)) or (‘Social-Networks’/all topical subheadings/all age subheadings in DE) or (explode ‘Counseling-’/all topical subheadings/all age subheadings in DE) or (‘Death-Education’/all topical subheadings/all age subheadings in DE) or (‘Patient-Education’/all topical subheadings/all age subheadings in DE) or (psychosocial in ab) or (psychosocial in ti)) and ((Clinical-Trial in DT) or (clinical-trials in DE) or (double-blind-studies in DE) or (single-blind-studies in DE) or (triple-blind-studies in DE) or ((singl^*^ or doubl^*^ or trebl^*^ or tripl^*^) and (mask^*^ or blind^*^)) or (‘latin square’) or (placebo in DE) or placebo^*^ or random^*^ or (study design in DE) or (explode ‘quasi experimental studies’/all topical subheadings/all age subheadings in DE) or (pretest posttest control group design in DE) or (solomon four group design in DE) or (crossover design in DE) or (repeated measures in DE) or (pretest posttest design in DE) or (experimental studies in DE) or control^*^ or prospective^*^ or volunteer^*^ or compar^*^)	((((‘Neoplasms-’ in DE) or (cancer in ab) or (tumor^*^ in ab) or (tumour^*^ in ab) or (malign^*^ in ab) or (oncolog^*^ in ab)) and ((‘Palliative-Care’ in DE) or ((‘Terminal-Cancer’ in DE) or (‘Terminally-Ill-Patients’ in DE)) or (‘Hospice-’ in DE) or (incurable in ab) or (incurable in ti) or (advanced in ab) or (advanced in ti) or (palliat^*^ in ab) or (palliat^*^ in ti) or (terminal^*^ in ab) or (terminal^*^ in ti))) and ((‘Coping-Behavior’ in DE) or (‘Support –Groups’ in DE) or (‘Social-Support-Networks’ in DE) or (explode ‘Psychotherapy –’ in DE) or (‘Cognitive-Therapy’ in DE) or (‘Art-Therapy’ in DE) or (‘Counseling-’ in DE) or (‘Self-Management’ in DE) or (‘Client-Education’ in DE) or (psychosocial in ab) or (psychosocial in ti))) and ((experimental design in DE) or (Clinical-Trial in pt) or (clinical trial) or ((singl^*^ or doubl^*^ or trebl^*^ or tripl^*^) and (mask^*^ or blind^*^)) or (‘latin square’) or (placebo in DE) or placebo^*^ or random^*^ or (follow-up studies in DE) or (prospective studies in DE) or (repeated-measures in DE) or (Treatment-Outcome-Study in pt) or (treatment effectiveness evaluation in DE) or control^*^ or prospective^*^ or volunteer^*^ or compar^*^)
Hits 328	Hits 179	Hits 77

). Second, abstracts of references of all relevant papers were retrieved and checked to identify additional studies. Third, to identify additional relevant studies the Science Citation Index was used to search for studies that have cited located, relevant papers. Fourth, leaders in the field were contacted to locate relevant but currently unpublished studies or suggest others who possibly know of unpublished work.

### Inclusion criteria

Retrieved studies were independently assessed for inclusion by two reviewers (RU and KP) and included if all of the inclusion criteria were met. Inclusion and exclusion criteria are summarised in Table 2

**Table 2:** Inclusion and exclusion criteria

*Inclusion criteria*:
A controlled study with a psychosocial intervention in at least one arm of the study
A study population of adult patients (⩾18 years of age) with cancer in an advanced stage of the disease (stage IV)
One (or more) dimension(s) of QoL should be at least one of the presented outcome measures

*Exclusion criteria*:
Studies concerning interventions that were not strictly psychosocial such as complementary therapies
Studies that used a comparison group other than usual care or attentional control group
Studies of which less than 50% of patients had cancer in an advanced stage of the disease

. Disagreement over inclusion between the reviewers was resolved through discussion. When no consensus could be achieved, a third researcher (TvA) decided.

Similar to previous reviews (Meyer and Mark, 1995; Barsevick *et al*, 2002) psychosocial interventions are defined to include counselling/psychotherapy, behaviour therapy, education and provision of information, social support or a combination of interventions. Quality of life was operationalised in global measures of QoL and measures concerning patient's emotional functioning (e.g. coping, mood state such as anxiety and depression or other type of emotional distress), social functioning, physical functioning (e.g. symptom distress, activity level, performance status, activities of daily living) and existential or spiritual concerns.

### Methodological quality

The adagium ‘garbage in–garbage out’ reveals that study quality is clearly relevant when conducting a systematic review. There is, however, limited empirical evidence of a relation between specific methodological quality criteria and bias, except for adequate concealment of treatment allocation and double blinding (van Tulder *et al*, 1997). Especially, the use of summary scores to identify studies of low or high quality is controversial (Moher *et al*, 1995; Jüni *et al*, 1999). Consequently, it is generally recommended to assess study quality against individual relevant methodological criteria, depending on the context in which studies are conducted, however, always including criteria concerning the internal validity of studies (Jüni *et al*, 1999; 2001). In this review the methodological quality of included studies was independently assessed by two reviewers (RU and ML) against nine criteria of internal validity (van Tulder *et al*, 1997). Each criterion was scored as yes, no or as providing insufficient information for adequate assessment. To ensure standardised scoring, a pilot-tested predesigned table was used. Disagreement among the reviewers was resolved by discussion.

### Data extraction

RU and ML independently extracted data. Predesigned tables were used to ensure that data extraction was standardised. Extracted information included: the sample (inclusion/exclusion criteria, type of cancer and disease stage), the setting (inpatient, outpatient, hospice and home-care setting), type of psychosocial intervention (counselling/psychotherapy, behaviour therapy, education and provision of information, social support and other psychosocial approaches), format of psychosocial intervention (group *vs* individual, structured *vs* unstructured, therapist, that is, psychotherapist or psychologist *vs* nurse delivered), time frame of psychosocial intervention (frequency, duration and follow-up), description of comparison group, nature of the outcomes measured (overall QoL, dimensions of QoL and other measured outcomes) and the study design. Disagreement among the reviewers was resolved by discussion.

Only measurements immediately post-treatment were included. When a study compared more than one intervention arm with the control arm, each intervention arm was labelled as a separate trial. It was envisioned that studies would be too heterogeneous to be combined using a formal meta-analysis. Therefore, a narrative synthesis was performed. The results are summarised according to type of intervention used and outcome measures assessed. The magnitudes of effects on each outcome measure are reported as the magnitudes of differences in change scores between groups, relative to the scale used.

## RESULTS

A total of 10 studies involving 13 trials were identified for inclusion in the review. The search of Medline, PsycInfo and Cinahl databases provided a total of 584 citations (Table 1). After adjusting for duplicates 509 remained. Of these, 479 studies were discarded because after reviewing the abstracts it appeared that these papers clearly did not meet the criteria. Three additional studies (Levy *et al*, 1990; Lee *et al*, 1997; Esper *et al*, 1999) were discarded because full text of the study was not available or the paper could not be feasibly translated into English. The full text of the remaining 27 citations was examined in more detail. It appeared that 22 studies did not meet the inclusion criteria as described (Table 3

**Table 3:** Excluded studies (*n*=22)

**Reason for exclusion**	**Studies**
Less than 50% of patients with advanced cancer	Decker *et al* (1992), Syrjala *et al* (1995), Johansson *et al* (1999), Oyama *et al* (2000), Carlson *et al* (2001), Lordick *et al* (2002) and Walker *et al* (1999a, b)
	
No controlled study design	Greer *et al*, 1991; Eysenck and Grossarth-Maticek, 1991; Bottomley, 1996; de Vries *et al*, 1997; Greer and Moorey, 1997; Cwikel and Behar, 1999; Gallagher and Steele, 2001)
	
Intervention not strictly psychosocial	Siegel *et al* (1992), Wilkinson *et al* (1999) and Ringdal *et al* (2001)
	
No QoL-related outcome measure	Sloman *et al* (1994) and Bruera *et al* (1999)
	
Comparison group other than usual care	Mantovani *et al* (1996) and Liossi and White (2001)

QoL=quality of life.

). Five studies (Connor, 1992; Corner *et al*, 1996; Scholten *et al*, 2001; Giese-Davis *et al*, 2002; Sloman, 2002) met the inclusion criteria and were included in the systematic review. An additional five studies (Arathuzik, 1994; Bredin *et al*, 1999; Edelman *et al*, 1999; Classen *et al*, 2001; Goodwin *et al*, 2001) that met the criteria for inclusion were identified by checking the references of located, relevant papers and searching for studies that have cited these papers. No unpublished relevant studies were obtained.

### Description of included trials

Characteristics of the included trials are shown in Table 4

**Table 4:** Characteristics of included trials (*n*=13)

			**Outcome^a^**		
			**Measures (instruments)^b^**	**Statistically significant**	
**Study**	**Participants**	**Interventions I=intervention and C= control**		***P*<0.05**	**Change score^c^**
Connor (1992) California, USA*Study quality*:Concealed treatment allocation: unclear Cointerventions: unclear Compliance: unclear Dropouts: 57%, *n* and reasons overall described, unclear if comparable between groups Intention to treat: no, patients lost clearly not in analysis	Terminally ambulatory ill cancer patientsExcluded if: Karnofsky <50 or cognitively impairedDisease stage: stage IV 100%Gender: 79% femaleMean age (range): 61 year (35–80 year)	I-group: counselling (*n*=13)*Content*: interview with patient, including questions about, for example, the most difficult aspects of having cancer? patients' believes about recovering from this illness and death?*Duration*: once*Setting*: inpatient*Format*: individual, structured, therapist-delivered,^d^ tailor-madeC-group: usual care (no intervention) (*n*=11)	*Emotional functioning*:Death anxiety (DAS)Denial (DMI)	NS+	—Small
					
Arathuzik (1994) Massachusetts, USA*Study quality*:Concealed treatment allocation: unclear Cointerventions: unclear Compliance: strict written protocol was followed to administer the intervention Compliance monitored: unclear Dropouts: unclear Intention to treat: unclear	Patients with confirmed metastatic breast cancer and experiencing physical painExcluded if: brain metastasis or terminal stageDisease stage: stage IV 100%Gender: 100% femaleAge range: 31–80 years (46%>60 years)	I-group 1: behaviour therapy – relaxation and visualisation (*n*=8)*Content*: discussion of a detailed description of; effects of relaxation on body and visualisation or mental images on the mind; specific breathing exercise, progressive muscle relaxation exercise and visualisation that would be practiced; conditions required to practise these exercises.Deep breathing exercise, relaxing muscle groups and visualisation exercises.*Duration*: once 75 min*Setting*: in-/outpatient*Format*: individual, structured, nurse-delivered, standardisedI-group 2: behaviour therapy - relaxation, visualisation and cognitive coping skills training (*n*=8); setting and format idem as I-group 1 *Content*: as I-group 1 adding to the discussion: a detailed description of the effects of distraction on pain and a review of written handouts on 23 methods of distraction.Instruction in specific positive affirmation such as ‘I can manage my pain..’Directed in practicing these affirmations and refocusing negative thoughts and feelings.*Duration*: once 120 minC-group: usual care (routine care and pain medication on an as needed basis) (*n*=8)	*Emotional functioning*:I-group 1: POMS subscalesI-group 1: ability to decrease pain (Rosenstiel)I-group 2: POMS subscalesI-group 2: ability to decrease pain (Rosenstiel)*Physical functioning*:I-group 1 and I-group 2: pain distress (Johnson Pain Distress scale)	NS+ NS+ NS	—Large — Moderate —
					
Corner *et al* (1996) London, UK*Study quality*:Concealed treatment allocation: unclear Cointerventions: unclear Compliance: unclear Dropouts: 41%, *n* and reasons described by group, comparable between groups Intention to treat: no, patients lost clearly not in analysis	Patients with non-small cell lung cancer who completed chemotherapy or radiotherapy suffering breathlessnessExcluded if: not mentionedDisease stage: stage IV 100%Gender: 40% femaleMedian age: Intervention group=55 yearsControl group=69 years	I-group: counselling and behaviour therapy (*n*=19)*Content*:Detailed assessment of patient's breathlessness, their disease and feelings about the future.Advice and support were given on methods of managing breathlessness and involving family members.Breathing retraining exercises.Goal setting to assist patient to learn breathing and relaxation techniques.*Duration*: weekly 1 h, 3–6 weeks*Setting*: outpatient*Format*: individual, structured, nurse-delivered, tailor-madeC-group: usual care (detailed assessment of breathlessness - no training or counselling) (*n*=15)	*Emotional functioning*:Anxiety (HADS)Depression (HADS)*Physical functioning*:Distress due to breathlessness (VAS)Ability to walk distances/climb stairs (Functional Capacity Scale)Difficulties performing ADL (checklist)	NS NS + + +	— — Large Small Small
					
Bredin *et al* (1999) London, UK*Study quality*:Concealed treatment allocation: yes Cointerventions: pharmacological interventions monitored and checked for balance Compliance: supervision, audits, using practice guideline to deliver the intervention Compliance monitored: yes, by completing checklist Dropouts: 41%, *n* and reasons described by group, not comparable between groups Intention to treat: yes	Patients with (non-) small cell lung cancer/mesothelioma who completed treatment and reporting breathlessnessExcluded if: not mentionedDisease stage: stage IV 100%Gender:Intervention group=female 20%Control group=female 33%Mean age (range):Intervention group=68 years (41–82 years)Control group=67 years (41–83 years)	I-group: counselling and behaviour therapy (*n*=52)*Content*: Detailed assessment of breathlessness and factors that ameliorate or exacerbate it.Advice and support for patients and their families on ways of managing breathlessness.Exploration of the meaning of breathlessness, their disease, and feelings about the future.Training in breathing control techniques, progressive muscle relaxation, and distraction exercises.Goal setting to complement breathing and relaxation techniques, to help in the management of functional and social activities and to support the development and adoption of coping strategies.Early recognition of problems warranting pharmacological or medical intervention.*Duration*: weekly, 3–8 weeks*Setting*: outpatient, nursing clinic*Format*: individual, structured, nurse-delivered and tailor-madeC-group: usual care (standard care, all patients had access to routinely available supportive care, that is, pharmacological, palliative treatments and treatment of associated problems such as anxiety or depression) (*n*=51)	*Emotional functioning*:Anxiety (HADS)Depression (HADS)Psychological symptom distress (RSCL)*Physical functioning*:Physical symptom distress (RSCL)Activity level (RSCL)Distress due to breathlessness (VAS)*Global measure of QoL*:QoL (RSCL)	NS + NS + + + NS	— Moderate — Large Moderate Large —
					
Edelman *et al* (1999) Sydney, Australia*Study quality*:Concealed treatment allocation: yes Cointerventions: participation in outside groups monitored and tested Compliance: unclear Dropouts: 25%, *n* and reasons described overall, comparable between groups Intention to treat: no, patients lost clearly not in analyses	Patients with metastatic breast cancerExcluded if: psychiatric or brain disorder; drug/alcohol dependencyDisease stage: stage IV 100%Gender: 100% femaleMean age (range): 50 years (29–65 years)	I-group: behaviour therapy (cognitive–behavioural therapy techniques) and social support (*n*=62)*Content*:At the start of the programme a manual was given.Handouts and homework exercises were given at every session. Basic cognitive skills were taught, including how to identify and challenge maladaptive thoughts and beliefs. Behavioural techniques were taught, for example, deep relaxation/meditation as a tool for managing anxiety. A relaxation tape was given to practise on a regular basis. Goal setting and problem solving to gain a greater sense of control.The first hour of each session was spent on discussing homework exercises, involving, for example, the recording of potentially stressful situations that arose during the week, identifying and disputing maladaptive thoughts and underlying beliefs. Participants were also asked to suggest ‘positive actions’ that they could take in order to resolve potentially problematic situations. In the second half of each session a particular theme was discussed. This was followed by discussion with participants reflecting on ways in which they could apply the discussed strategies to their own particular circumstances and experiences.*Duration*: weekly, 8 weeks*Setting*: outpatient*Format*: group, structured, therapist-delivered and tailor-madeC-group: usual care (standard oncological care) (*n*=62)	*Emotional functioning*:Anxiety (POMS)Depression (POMS) Anger (POMS) Fatigue (POMS) Confusion (POMS) Vigour (POMS) Total Mood Diaturbance (POMS)Self-esteem (Coopersmith Self Esteem Inventory)	NS + NS NS NS NS + +	— Small — — — — Small Small
					
Scholten *et al* (2001) Austria*Study quality*:Concealed treatment allocation: unclear Cointerventions: unclear Compliance: unclear Dropouts: unclear Intention to treat: unclear	Patients with metastatic breast cancerExcluded if: central nervous system metastasisDisease stage: stage IV 100%Gender: 100% femaleMean age (s.d.):Intervention group=61.6 years (8.7 years)Control group=62.2 years (8.1 years)	I-group: behaviour therapy – cognitive and behavioural approaches (*n*=20)*Content*:Strategies as problem solving, regaining control, setting new goals for the future.Therapy focused on patients' coping strategies, self-esteem and femininity, overcoming feeling helpless, negative thoughts and depression, and promotion of a fighting spirit.For symptom control, behavioural techniques (exercises in self-hypnosis and progressive muscle relaxation) were employed.*Duration*: 6 months*Setting*: outpatient*Format*: individual, structured, therapist-delivered and tailor-madeC-group: usual care (patients received no psychosocial support during observation period) (*n*=23)	*Emotional functioning*: Cognitive, emotional and behavioural coping skills (semi-structured interviews) *Global measures of QoL*: (Non-) health related quality of life (VAS)	NS NS	— —
					
Classen *et al* (2001) California, USA*Study quality*:Concealed treatment allocation: yes Cointerventions: all patients were offered self-directed education intervention, use was monitored and checked for balance. Compliance: supervision, use of protocol to deliver intervention Compliance monitored: unclear Dropouts: 18%, *n* and reasons described by group, acceptable Intention to treat: yes (for patients with at least one follow-up measurement)	Patients with confirmed metastatic or recurrent breast cancerExcluded if: Karnofsky score <70 or no metastasis beyond supraclavicular nodesDisease stage: stage IV 100%Gender: 100% femaleMean age (s.d.): Intervention group=54.0 years (10.7 years)Control group=52.9 years (10.7 years)	I-group: behaviour therapy (cognitive–behavioural approaches) and social support (*n*=58)*Content*: Supportive Expressive Therapy (SET). Therapists were trained to facilitate discussion in an emotionally expressive rather than a didactic format of the following themes:Fears of dying and death including dealing with death of group members.Reordering life priorities.Improving support from and communication with family and friends.Integrating a changed self- and body image.Improving communication with physicians.Learning self-hypnosis for pain an anxiety control.Through sharing of their experiences, group members became role models for one another. Leaders kept members focused on issues central to their diagnoses of metastatic breast cancer and on facing and grieving for their losses*Duration*: weekly 1.5 h for 1 year*Setting*: outpatient*Format*: group, unstructured, therapist-delivered, tailor-madeC-group: usual care (no specific description) (*n*=44)	*Emotional functioning*:Total Mood Disturbance (POMS)Intrusion and avoidance – total score (IES)	+ +	Small Small
					
Goodwin *et al* (2001) Canada*Study quality*: Concealed treatment allocation: yes Cointerventions: all patients received educational materials every 4–6 months. Unclear if use was monitored. Participation in support groups was monitored and proved comparable between groups. Compliance: use of protocol, every 9–12 months workshops, review of randomly selected videotaped sessions. Compliance monitored: unclear Dropouts: 34%, *n* and reasons described overall, comparable between groups Intention to treat: yes	Patients with confirmed metastatic breast cancerExcluded if: life expectancy <3 months; no metastasis beyond ipsilateral axilla; central nervous system metastasis; untreated major depression; planned participation in therapist-led support group for patients with metastatic breast cancer outside the study centerDisease stage: stage IV 100%Gender: 100% femaleMean age (s.d.): Intervention group=49.5 years (8.4 years)Control group=51.5 years (10.3 years)	I-group: behaviour therapy (cognitive–behavioural approaches) and social support (*n*=158)*Content*: SET – see Classen (2001)*Duration*: 90 min weekly, 1 year*Setting*: outpatient*Format*: group, unstructured, nurse- and therapist-delivered, tailor-madeC-group: usual care (patients did not receive any psychological therapy as part of the study but could however participate in peer support groups or therapist led support groups that did not involve SET) (*n*=77)	*Emotional functioning*:Anxiety (POMS)Depression (POMS)Anger (POMS)Fatigue (POMS)Confusion (POMS)Vigour (POMS)Total Mood Disturbance (POMS)*Physical functioning*:Experience of suffering	+ + + NS + NS + NS	Small Small Small — Small — Small —
					
Giese-Davis *et al* (2002) California, USA*Study quality*: Concealed treatment allocation: yes Cointerventions: all patients were offered educational materials and 1-year membership of a health library. Unclear if use was monitored. Compliance: ongoing supervision, use of protocol Compliance monitored: unclear Dropouts: 42%, *n* and reasons described overall, not comparable between groups Intention to treat: yes, for patients with at least one follow-up measurement	Patients with documented metastatic or recurrent breast cancerExcluded if: Karnofsky score <70 or no metastasis beyond supraclavicular nodes Disease stage: stage IV 100%Gender: 100% femaleMean age (s.d.): Intervention group=52.7 years (10.5 years)Control group=53.8 years (10.5 years)	I-group: behaviour therapy (cognitive–behavioural approaches) and social support (*n*=64)*Content*: SET – see Classen (2001)*Duration*: weekly 1.5 h for 1 year*Setting*: outpatient*Format*: group, unstructured, therapist delivered, tailor-madeC-group: usual care (no specific description) (*n*=61)	*Emotional functioning*:Anger control (CECS)Depression control (CECS)Anxiety control (CECS)Total score (CECS)Restraint (WAI)Defensiveness (WAI)Confidence communicating emotions (SESES-C)Confidence confronting death (SESES-C)Confidence focusing on present moment (SESES-C)Emotional self-efficacy total score (SESES-C)	+ + NS + + NS NS NS + NS	NE^e^ NE^e^ NE^e^ Small Small — — — NE^e^ —
					
Sloman (2002) Israel*Study quality*:Concealed treatment allocation: unclearCointerventions: unclear Compliance: unclear Dropouts: unclear Intention to treat: unclear	Ambulatory patients with advanced cancer experiencing anxiety and depression without ever been trained in progressive muscle relaxation (PMR) or guided imagery (GI) Excluded if: not mentionedDisease stage: stage IV 63%Gender: 46% femaleMean age: 54.4 years (range 27–79 years)	I-group 1: behaviour therapy - progressive muscle relaxation training (*n*=14)*Content*: Nurse explained the nature of the session and turned on the taped instructions. At the end the nurse had a brief discussion concerning any problems that patients may have experienced in following the instructions.The tape recorder was left to practice PMR technique twice daily. Making of follow-up appointments for the nurse to visit subjects twice weekly to repeat the sessions and deal with any related problems.Cassette tapes contained clear instructions by a clinical psychologist that guided the subjects in the use of the techniques.*Duration*: 30 min, twice weekly, 3 weeks	*Emotional functioning*:I-group 1: Anxiety (HADS)Depression (HADS)I-group 2: Anxiety (HADS)Depression (HADS)I-group 3: Anxiety (HADS)Depression (HADS)*Global measures of QoL*:I-group 1: Functional Living Index Cancer Scale (FLIC)I-group 2: Functional Living Index Cancer Scale (FLIC)I-group 3: Functional Living Index Cancer Scale (FLIC)	NS + NS + NS + + + +	— Small — Small — SmallSmall Small Small
		*Setting*: home care*Format*: individual, structured, nurse delivered, standardizedI-group 2: behaviour therapy-guided imagery; content, duration, setting and format idem as I-group 1 but focus on GI (*n*=14)I-group 3: behaviour therapy−PMR+GI; content, duration, setting and format idem as I-group 1, but focus on PMR training+GI (*n*=14)C-group: usual care (no relaxation or imagery training; to control for placebo effect a nurse spent an equal amount of contact time with control subjects and had a general discussion about their concerns relating to their health, nursing care, and medical treatments) (*n*=14)			

QoL=quality of life; s.d.=standard deviation; NS=nonsignificant.

a Outcomes outside the scope of this review are not presented.

b POMS=Profile of Moods Scale; HADS=Hospital Anxiety and Depression Scale; RSCL=Rotterdam Symptom Checklist; IES=Impact of Event Scale; DAS=Death Anxiety Scale; DMI= Defense Mechanisms Inventory; CECS=Courtauld Emotional Control Scale; WAI=Weinburger Adjustment Inventory; SESES-C= Stanford Emotional Self-Efficacy Scale - Cancer; FLIC= Functional Living Index-Cancer Scale.

c Magnitude of the difference in change scores between groups relative to the scale used – small=change score <25%; moderate=change score between 25 and 50%; large=change score >50%.

d When therapist is written psychotherapist and psychologist are meant.

e NE=not evaluable, in this trial slopes analysis was used to measure change over time and only mean change of slope was given for this measure.

. All 13 included trials used a randomisation procedure to allocate the psychosocial intervention. Included trials were predominantly conducted in Europe (*n*=3 trials) and the United States of America and Canada (*n*=6).

#### Sample characteristics

The mean sample size of the intervention and control group was 39 (range 8–158 patients) and 31 patients (range 8–77 patients), respectively. The average age of patients ranged from 50 to 67 years. In eight trials, the majority of patients were female. Seven trials included only female patients and concerned patients with breast cancer. Six trials recruited patients with cancer at any site. Three trials (Connor, 1992; Corner *et al*, 1996; Bredin *et al*, 1999) limited the inclusion to patients who were clearly in far advanced stages of the disease. The mean percentage of patients with advanced disease (stage IV) was 89% (range 63–100%).

#### Setting

The majority of the trials were conducted in an outpatient (*n*=7 trials) or home-care setting (*n*=3). Only one trial was conducted in an in-patient setting. Two trials were conducted in a combination of in- and outpatient settings.

#### Type of intervention

The content of the experimental psychosocial interventions was quite different. However, behaviour therapy was used in 12 trials, including one or more of the following: relaxation exercises, guided imagery, visualisation or cognitive approaches focusing on changing specific thoughts or beliefs and learning specific coping skills. In six (Arathuzik, 1994; Scholten *et al*, 2001; Sloman, 2002) of these 12 trials, behaviour therapy was used as a single intervention. A combined intervention of behaviour therapy and group support was used in four trials (Edelman *et al*, 1999; Goodwin *et al*, 2001; Classen *et al*, 2001; Giese-Davis *et al*, 2002). The group-support intervention in these trials involved the creation of a supportive environment in which patients were encouraged to express their emotions about cancer and its broad-ranging effects on their lives. Patients were also encouraged to interact with and support each other. Practical or structural aspects of social support were not covered in these studies. Two trials (Corner *et al*, 1996; Bredin *et al*, 1999) combined behaviour therapy with counselling and involved training in breathing control techniques, relaxation and distraction exercises. In addition, the meaning of breathlessness, their disease and patients' feelings about the future were explored. Counselling was used as a single intervention in one trial. In this trial (Connor, 1992), terminally ill ambulatory patients were interviewed to create an opportunity for the patient to explore and to gain insight into his own coping processes. The interview was paced so that those who gave guarded responses were not required to elaborate. Subjects who were less guarded were encouraged to talk more about their feelings, perceptions and memories.

#### Format of the intervention

Four trials (Edelman *et al*, 1999; Classen *et al*, 2001; Goodwin *et al*, 2001; Giese-Davis *et al*, 2002) delivered the intervention in a group format. Nurses or both nurses and therapists, that is, psychotherapists or psychologists, delivered the intervention in a majority of trials (*n*=8). In eight trials interventions were tailor-made to the needs and preferences of included patients, whereas the intervention in five trials was standardised. A total of 10 trials involved a multisession intervention, of which four trials (Edelman *et al*, 1999; Classen *et al*, 2001; Goodwin *et al*, 2001; Giese-Davis *et al*, 2002) delivered the intervention during a period of 8 weeks or longer (up to 1 year).

#### Outcome measures

Outcome measures to investigate the effects of the interventions were all questionnaire based except in one trial (Scholten *et al*, 2001), which additionally used semistructured interviews.

Outcome measures were strongly heterogeneous, especially coping measures, measures of physical functioning and global measures of QoL.

Five different measures of emotional distress were used across 11 trials. The Profile of Mood States (McNair *et al*, 1992) and the Hospital Anxiety and Depression Scale (Zigmond and Snaith, 1983) were the most frequently used (*n*=5 trials) measures. The Rotterdam Symptom Checklist, psychological symptom distress subscale (De Haes *et al*, 1996), was used in the trial of Bredin *et al* (1999). In one trial (Edelman *et al*, 1999), the Coopersmith Self-Esteem Inventory (Myhill and Lorr, 1978) was used. In another trial (Connor, 1992), death anxiety was measured using a scale of the same name (McMordie, 1979).

Seven different measures of coping were used across six trials. The Courtauld Emotional Control Scale (Watson and Greer, 1983), Weinburger Adjustment Inventory (Weinberger, 1997) and Stanford Emotional Self-Efficay Scale – Cancer (Giese-Davis *et al*, 2002) were used in a trial (Giese-Davis *et al*, 2002) to, respectively, measure the extent to which patients report they suppress negative affect, restrain from aggressive behaviour and are emotional self-efficacious. Patients' perceived ability to decrease or control pain (Rosenstiel and Keefe, 1983) was measured in two trials (Arathuzik, 1994). In one trial (Connor, 1992), the use of denial was measured by the Defense Mechanism Inventory (Gleser and Sacks, 1973). In another trial (Classen *et al*, 2001), the Impact of Event Scale (IES) (Sundin and Horowitz, 2002) was used to measure symptoms of intrusion and avoidance. Semistructured interviews to measure coping skills were used once (Scholten *et al*, 2001).

Physical functioning was measured across five trials by six different measures of physical functioning, varying from the Rotterdam Symptom Checklist, physical symptom distress subscale (De Haes *et al*, 1996) to a visual analogue scale measuring distress due to breathlessness (Corner *et al*, 1996; Bredin *et al*, 1999).

Four different global measures of QoL, for example, the Rotterdam Symptom Checklist subscale – overall evaluation of QoL and the Functional Living Index – cancer scale, were used across five trials.

### Methodological quality of included trials

The methodological quality of the included trials is summarised in Table 5

**Table 5:** Methodological quality of included trials (*n*=13)

	**No. of trials (%)**		
**Quality indicator**	**Yes, fulfilled**	**No**	**Unclear**
Method of randomisation	13 (100%)	—	—
Adequate concealment of allocation	5 (38%)	—	8 (62%)
			
*Groups similar at baseline*:			
Gender	10 (77%)	—	3 (23%)
Age	6 (46%)	3 (23%)	4 (31%)
Functional status	6 (46%)	1 (8%)	6 (46%)
Emotional distress	8 (62%)	1 (8%)	4 (31%)
Key outcome measures	9 (69%)	1 (8%)	3 (23%)
			
*Cointerventions*:			
Cointerventions avoided by trial design	3 (23%)	10 (77%)	—
Cointerventions monitored	4 (31%)	—	9 (69%)
			
Compliance monitored and acceptable	6 (46%)	—	7 (54%)
			
Blinded outcome assessment for at least one key outcome	—	13 (100%)	—
			
Relevant outcome measures	13 (100%)	—	—
			
*Withdrawal/dropout rate*:			
*N* and reasons described	7 (54%)	—	6 (46%)
% lost ⩽20% or comparable between groups	4 (31%)	2 (15%)	7 (54%)
			
Comparable timing of outcome assessment in both groups	13 (100%)	—	—
			
Intention-to-treat analyses explicitly stated	4 (31%)	3 (23%)	6 (46%)

. In five trials (Bredin *et al*, 1999; Edelman *et al*, 1999; Classen *et al*, 2001; Goodwin *et al*, 2001; Giese-Davis *et al*, 2002), treatment allocation was adequately concealed. This was either carried out through central off-site randomisation, block randomisation or an adaptive randomisation-biased coin design. In eight trials, insufficient information was available to assess if concealment of treatment allocation was adequately performed.

The number of trials that had similar groups at baseline with regard to the most important prognostic indicators ranged from six trials for age and functional status to 10 trials for gender. Nine trials had similar groups at baseline for the key outcome measures (see Table 5, Section 2). One trial (Corner *et al*, 1996) reported that groups were not similar at baseline, that is, patients in the intervention group had higher distress caused by breathlessness, higher anxiety levels and greater difficulty performing activities of daily living.

All 13 trials gave a relatively detailed description of the intervention. With regard to care in the control group, nine trials explicitly mentioned patients received usual, standard or routine care. Five of these trials (Bredin *et al*, 1999; Goodwin *et al*, 2001; Sloman, 2002) gave a fairly detailed description of this type of care.

Avoidance of cointerventions by trial design or monitoring of the use of cointerventions was mentioned in five trials. Concerning avoidance of cointerventions by trial design, three trials (Classen *et al*, 2001; Goodwin *et al*, 2001; Giese-Davis *et al*, 2002) controlled for imbalance in the use of information by offering educational materials to patients in both the intervention and control group. Of which, the trial of Classen *et al* (2001) also mentioned the monitoring of the use of these educational materials. Participation of patients in outside support groups was monitored by Edelman *et al* (1999) and Goodwin *et al* (2001). Bredin *et al* (1999) monitored the use of pharmacological interventions.

One trial (Bredin *et al*, 1999) explicitly mentioned the monitoring of compliance. Six trials (Arathuzik, 1994; Bredin *et al*, 1999; Classen *et al*, 2001; Goodwin *et al*, 2001; Giese-Davis *et al*, 2002) mentioned the use of a protocol, supervision, reviews of videotaped intervention sessions or side visits to ensure that the intervention was delivered as intended.

The withdrawal/dropout of patients was described in seven trials; three trials (Corner *et al*, 1996; Bredin *et al*, 1999; Classen *et al*, 2001) described number and reasons for dropout by group and four trials (Connor, 1992; Edelman *et al*, 1999; Goodwin *et al*, 2001; Giese-Davis *et al*, 2002) described this for the total sample. The drop-out rate was considered acceptable when 20% or less (Classen *et al*, 2001) or comparable between groups (Corner *et al*, 1996; Edelman *et al*, 1999; Goodwin *et al*, 2001). The dropout rate ranged from 18 to 57%.

Approximately one-third of the trials (Bredin *et al*, 1999; Classen *et al*, 2001; Goodwin *et al*, 2001; Giese-Davis *et al*, 2002) explicitly stated that intention-to-treat analyses were used to deal with patients who were lost to follow-up. However, two trials (Classen *et al*, 2001; Giese-Davis *et al*, 2002) limited this intention-to-treat analyses to patients with at least one follow-up measurement. Three trials (Connor, 1992; Corner *et al*, 1996; Edelman *et al*, 1999) did not use intention-to-treat analyses, that is, patients lost to follow-up were clearly not in the analyses.

Parametrical statistical tests were used in 10 trials (Connor, 1992; Arathuzik, 1994; Edelman *et al*, 1999; Classen *et al*, 2001; Goodwin *et al*, 2001; Giese-Davis *et al*, 2002; Sloman, 2002). Nonparametrical tests were used in three trials (Corner *et al*, 1996; Bredin *et al*, 1999; Scholten *et al*, 2001). Eight trials compared four or more outcome measures. All trials used a *P*-value of 0.05 as the limit of statistical significance.

### Effects on QoL

Outcomes of trials that aimed at improving one of the dimensions of patients' QoL are summarised in Table 4.

#### Emotional functioning – distress

Anxiety and depression as measures of emotional functioning were used in 10 trials (Arathuzik, 1994; Corner *et al*, 1996; Bredin *et al*, 1999; Edelman *et al*, 1999; Classen *et al*, 2001; Goodwin *et al*, 2001; Sloman, 2002). One trial (Goodwin *et al*, 2001) showed a statistically significant treatment effect for anxiety. Whereas in six trials (Sloman *et al*, 2002; Bredin *et al*, 1999; Edelman *et al*, 1999; Goodwin *et al*, 2001), a statistically significant treatment effect for depression was found. Patients' self-esteem improved significantly following the combined intervention of behaviour therapy and group support (Edelman *et al*, 1999). No significant effect was found for death anxiety as an outcome of an individual counselling intervention in a sample of ambulatory terminally ill patients (Connor, 1992).

#### Emotional functioning – coping

In five of the six trials (Connor, 1992; Arathuzik, 1994; Classen *et al*, 2001; Scholten *et al*, 2001; Giese-Davis *et al*, 2002) in which patients' coping abilities were measured, a significant treatment effect was found. One of these trials (Giese-Davis *et al*, 2002) investigated the effects of supportive-expressive group therapy on emotion-regulation outcome measures and found a significant reduction in suppression of feelings of anger, sadness and fear while also showing a significant improvement in greater restraint of aggressive, inconsiderate, irresponsible and impulsive behaviour. Furthermore, patients in the treatment group reported that they were significantly better able to focus on the present. Another trial (Classen *et al*, 2001) also investigating supportive-expressive group therapy showed a significant reduction in total scores on IES, measuring symptoms of intrusion and avoidance. Two trials (Arathuzik, 1994) examining individual behavioural therapy showed a significant improvement of patients' perceived ability to decrease/control their pain. One trial (Connor, 1992) found a significant reduction in the use of denial by patients receiving individual counselling compared to patients in the control group.

#### Physical functioning

Positive effects were seen in two of five trials (Arathuzik, 1994; Corner *et al*, 1996; Bredin *et al*, 1999; Goodwin *et al*, 2001) measuring physical functioning. Both trials (Corner *et al*, 1996; Bredin *et al*, 1999) investigated the effects of a nursing intervention for breathlessness in patients with lung cancer involving a combination of individual behaviour therapy and counselling, and found a significant improvement for physical symptom distress, activity level and functional capacity.

#### Global measures of QoL

Significant improvements of QoL were found in three of the five trials (Bredin *et al*, 1999; Scholten *et al*, 2001; Sloman, 2002) measuring overall QoL. More specifically, results showed an improvement of scores on the Functional Living Index – cancer scale (Sloman, 2002).

## DISCUSSION

### Main results

Evidence to date indicated that psychosocial interventions pertaining to the field of behaviour therapy were beneficial for patients with advanced cancer. Of 13 included trials, 12 showed positive effects on one or more indicators of QoL. The main benefit is an improvement of depression and feelings of sadness.

Coping also improved, especially a reduction in suppression of negative affect and an improvement in the restraint of inconsiderate and impulsive behaviour. As most effects were of a small magnitude, it is unclear if these effects are of clinical significance. Yet, as the effects reflected changes on measures that are important to patients with advanced cancer, that is, alleviating distress or improving patient's (perceived) functioning in daily life, even small effects can be clinically significant.

This review illustrated that the most frequently used type of psychosocial interventions for patients with advanced cancer was behaviour therapy as a single or combined intervention. Behaviour therapy was associated with cognitive-behavioural techniques and is an approach used to modify behaviour. This approach is based on the belief that cognitions prompt and mediate behaviour and that they are amenable to change. Behaviour therapy intends to help patients acquire strategies to better manage their behaviour, for example, increasing such skills as voluntary relaxation of the skeletal muscles, diverting attention away from pain or other distressing symptoms, reinterpreting pain or other distressing symptoms by changing unhelpful thoughts and believes, and developing a view of oneself that emphasises a sense of mastery of control and self-reinforcement of adaptive behaviour.

The review showed that therapists, that is, psychotherapist or psychologists, nurses or nurses and therapists combined, delivered behaviour therapy. It is worth noting that the interventions given by a therapist were tailor-made, predominantly with a group format and taking longer than 8 weeks, whereas interventions delivered by nurses or nurses and therapists combined were predominantly standardised, individual and given over an 8-week period or shorter. This review, however, does not answer the question whether certain characteristics of the intervention, for example, shorter or longer duration, an individual or group format, standardised or tailor-made and delivered by either therapists, nurses or nurse and therapist combined, might influence the effect, as it was not possible to perform a meta-analysis and subgroup analyses because of the heterogeneity of the data.

Furthermore, the results of the individual trials illustrate that outcome measures pertaining to the QoL dimension of emotional functioning, that is, anxiety and depression, were most frequently measured. Physical functioning and global measures of QoL were less frequently used. Similarly, measures of social functioning were not used. In addition, variables concerning the spiritual or existential domain of QoL were never measured. Especially in the case of patients with advanced cancer, it appeared that this domain is of great importance to patients and should not be ignored (Cohen *et al*, 1996; Rinck *et al*, 1997; Skeel, 1998).

### Limitations

This review was conducted rigorously and provides a balanced assessment of the current evidence. The search was extensive and it is unlikely that controlled trials were missed. Efforts have been made to identify unpublished studies. Despite this the review may be subject to publication bias, although we did not find that trials reporting beneficial results were the methodological weaker trials. A formal method of assessing publication bias, for example, funnel plots, could, however, not be performed because of great heterogeneity among the trials.

The conclusions that can be drawn from this review should be treated with some caution, because of the limitations in the evidence. There were problems with the validity of the studies. Limited details of the methods used in the trial, including methods of randomisation, monitoring of the use of cointerventions and compliance, were available. Especially in trials with advanced cancer patients, where care needs and preferences of patients vary greatly and where because of this cointerventions cannot be avoided, it is of great importance that all cointerventions are monitored and checked for imbalance between groups (Rinck *et al*, 1997). The same holds true for the monitoring of compliance. Although blinding of the outcome assessment is undoubtedly an important methodological quality indicator, in trials where outcome measures are self-reported and patients know which group they were allocated to, this is clearly impossible. Another limitation concerns the attrition of patients. In trials with advanced cancer, patients' untimely attrition must be expected and accounted for; however, limited details concerning patient attrition were available in about half of the included trials. A further limitation concerns the similarity of groups at baseline for key outcome measures. One trial reported an imbalance in baseline values and possibly affected the outcomes of physical functioning in this particular trial. Another limitation involves the statistical power of the trials. Some trials were clearly underpowered. On the other hand, it appeared that even these underpowered trials showed statistical significant changes, which possibly could have achieved the level of clear clinical significance when adequately powered. Also, limited details concerning the appropriateness of statistical analyses were given. Another limitation concerns the multiple comparisons that were made in most of the trials without adjusting their limit of statistical significance. However, to elude this ‘data-dredging’ phenomenon that possibly occurred in some of the trials, relevant outcome measures for this review were *a priori* selected.

The generalisability of the results from the included trials is questionable to the extent that performance status and life expectancy criteria applied in the majority of the studies will have led to the selection of patients who may have been somewhat healthier than those who would be offered psychosocial intervention in practice.

## CONCLUSION

In summary, there is an indication that psychosocial intervention using cognitive-behavioural techniques are beneficial for the QoL of patients with advanced cancer, especially in the domain of emotional functioning. However, evidence is limited as there have been few large methodological strong trials. On the other hand, it is nearly impossible that all methodological quality criteria will be met when conducting a trial in the field of palliative care. Undoubtedly, scientific rigour should always be aimed at. However, at the same time one needs to consider that given the circumstances of providing care to patients with advanced cancer, this demand resembles a mathematical asymptotic function, where regardless of effort total rigour cannot be achieved. As nearly all included trials focused on behaviour therapy, nothing can be inferred about the effectiveness of nonbehaviour therapy techniques.

Based on the results and above consideration, the authors of this review conclude that practitioners and health educators should consider an intervention involving techniques of behaviour therapy to address emotional distress in patients with advanced cancer. It remains unclear if a particular format of behaviour therapy is more beneficial than others. More research testing the effects of psychosocial interventions in patients with advanced cancer is needed. For future research, it is recommended to (also) involve outcome measures pertaining to the existential/spiritual domain of QoL, to address the issues of clinical significance and statistical power, to provide information in sufficient detail making methodological assessment possible, to form comparable groups on key outcome measures and to monitor the use of cointerventions and compliance.
